# Inset Guide for the Osteocutaneous Fibula Flap with Endosseous Implants in Oncologic Jaw Reconstruction

**DOI:** 10.1097/GOX.0000000000002475

**Published:** 2019-10-10

**Authors:** Evan B. Rosen, Robert J. Allen, Jonas Nelson, Evan Matros

**Affiliations:** From the Department of Surgery, Memorial Sloan Kettering Cancer Center, New York, N.Y.

## Abstract

Reconstruction of segmental maxillary or mandibular defects with osteocutaneous free flaps can be reliably accomplished; however, buccal or lingual rotation of the fibula during rigid fixation can render immediate endosseous implant position unusable for functional dental rehabilitation. To address this issue, a custom inset guide is introduced which utilizes surface topography of the immediately placed dental implant abutments and the patient’s dentition to orient the fibula segments during inset. Use of this technique facilitates successful endosseous implant position to optimize postoperative functional rehabilitation.

## INTRODUCTION

Reconstruction of segmental maxillary or mandibular defects can be reliably accomplished using osteocutaneous free flaps, most commonly the fibula. In addition to the esthetic and functional benefits of this approach, osteocutaneous free flaps are a suitable substrate for endosseous implants to restore the dentition removed as part of tumor ablation.^1,2^ Delayed endosseous implant placement, the most common scenario, prolongs complete functional recovery and often requires secondary procedures to remove fixation hardware screws coinciding with implant placement. In contrast, immediate implant placement into the fibula can mitigate interference with fixation hardware; however, one potential drawback of this approach is that it does not ensure either accurate or precise implant position.^3^

**Fig. 1.**
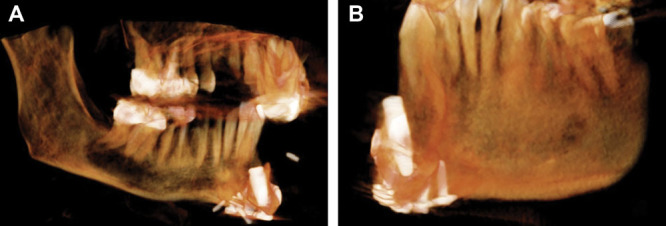
Malpositioned fibula segments following inset because of movement from the preoperative plan. Effect is exaggerated demonstrating (A) lingual and (B) buccal oriented endosseous implants originally planned to be vertical to the occlusal plane.

If immediate endosseous implant placement is desired, fibula positioning during insetting is critical as it impacts future oral rehabilitation. When the trajectory of the endosseous implants deviates from the occlusal plane by an extreme degree (ie, >30 degrees), then placement of the intraoral prosthesis by the prosthodontist or dentist may not be salvageable. In our experience, malpositioning of the fibula segment has largely been the result of lingual or buccal rotation of the fibula flap during rigid fixation (Fig. 1). Lack of access to virtual surgical planning, primarily due to cost, may exaggerate the severity of the malpositioned fibula segment. To ensure accuracy and precision according to the virtual surgical plan and computer-aided design, a custom fabricated inset guide has been designed as follows.

## TECHNIQUE

**Fig. 2.**
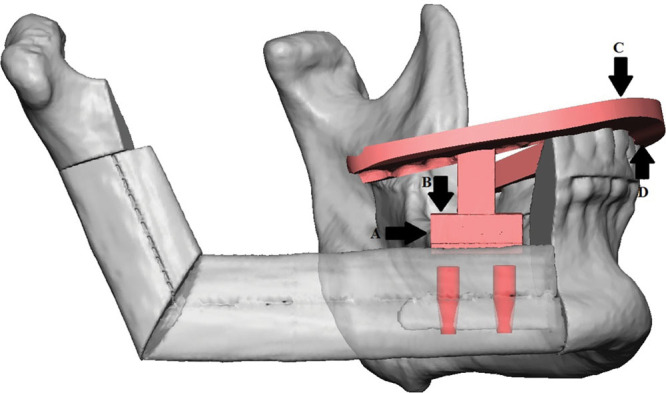
Inset guide for osteocutaneous free flap with endosseous implants designed using virtual surgical planning and computer-aided design and manufacturing (Courtesy of 3D Systems Healthcare, Littleton, Colo.). Four points of registration indicated: lateral portion of implant abutment (A), superior portion of implant abutment (B), incisal edges of maxillary dentition (C), and incisal edges of mandibular dentition (D).

**Fig. 3.**
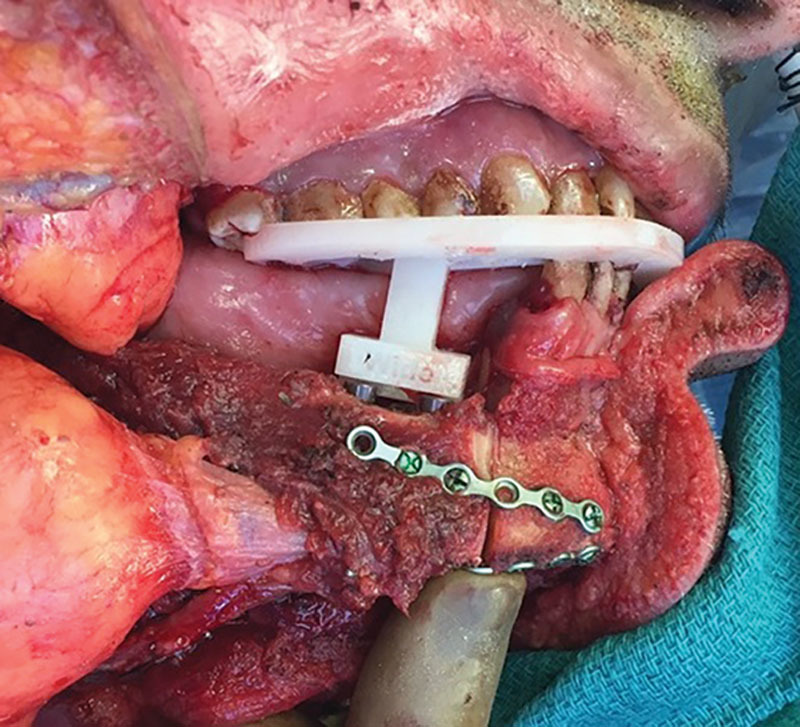
Rigid fixation completed with the inset guide in place.

The inset guide comprised 4 different registration areas that allow reliable placement in the oral cavity. The occlusal portion of the guide has registration points for the maxillary and mandibular dentition. This requires the patient to be in full occlusion during inset. To fabricate this portion of the guide, an accurate digital model of the patient’s occlusion must be provided. Often CT data of the occlusion is unusable due to dental artifact that obscures this portion of the imaging. Dental models or intraoral scanning can be used as an alternative to supplement the CT and provide high-resolution tooth surface detail to correct this issue. The occlusal portion of the guide then connects to the defect area with a vertical strut that has 2 walls on which to position the fibula segment. As there are endosseous implants in the fibula free flap, the abutments (components that are tightened into the superior aspect of the implants) are of known geometries and can be used as topographic registration points. This allows the guide to register on exposed metallic elements instead of the fibula which may be covered with periosteum and thus more challenging to properly position. We have found that the superior and lateral (lingual) portions of the abutments are favorable surfaces on which to register the guide so visibility during fixation is not obstructed (Fig. 2). These locations ensure that the fibula segment is not rotated and is in the correct vertical position. Additionally, the superior and lateral portions of the guide are not restrictive so that placement of the fibula segment into the correct orientation can quickly be accomplished. This strategy for registration can be used with any implant system; however, the implant abutment geometry may differ depending upon the abutment used and must be considered during guide design. Rigid fixation is then completed with the guide in place using the surgeon’s preferred plating system (Fig. 3).

## DISCUSSION

Minor or moderate amounts of fibula malrotation during inset into the mandible are generally not a critical success factor for reconstruction because it is an inconsequential subclinical event. However, as more aspects of jaw reconstruction such as dental restoration are performed immediately, this becomes an important detail to consider. Rotation of the fibula has been identified as a source of error and observed to occur for a number of reasons. First, the absence of dentition on the fibula segment does not allow for observations of occlusal positioning with the corresponding mandible or maxillary dentition. In essence, the surgeon has no choice, but to guess. The “Jaw in a Day,” surgery, commonly used for benign pathology, is able to overcome this limitation because it connects prosthetic teeth to endosseous implants with immediate evaluation of the occlusion.^4^ However, in malignant cases, when a skin island is needed for oral cavity lining replacement, perforation of the skin island to connect prosthetic teeth to endosseous implants is undesirable. Second, methods of fixation such as reconstruction plates would seemingly be helpful to ensure reliable positioning of the fibula segment, yet fixation of the flap promotes adaptation to the reconstruction plate rather than to what is needed for the occlusion. To counter the restrictions or limited degrees of freedom imposed by reconstruction plates, our group often substitutes these larger heavy plates for miniplates which allow for more nuanced inset.

## CONCLUSIONS

The opportunity to offer patients with malignant pathology immediate implant placement answers an unmet need but has created additional challenges. The creation of a custom inset guide has proven to be very helpful during fibula inset to predictably position segments in the correct orientation and reduce the opportunity for malpositioning of endosseous implants. Limitations of this technique are physician access to computer-aided design and manufacturing software for inset guide fabrication. Access to a dental team to obtain intraoral teeth scans or dental models is also a possible limitation.
